# Grid-Based Mobile Robot Path Planning Using Aging-Based Ant Colony Optimization Algorithm in Static and Dynamic Environments

**DOI:** 10.3390/s20071880

**Published:** 2020-03-28

**Authors:** Fatin Hassan Ajeil, Ibraheem Kasim Ibraheem, Ahmad Taher Azar, Amjad J. Humaidi

**Affiliations:** 1 Department of Electrical Engineering, College of Engineering, University of Baghdad, Baghdad 10001, Iraq; fatin.hassan0@gmail.com (F.H.A.); ibraheemki@coeng.uobaghdad.edu.iq (I.K.I.); 2 Robotics and Internet-of-Things Lab (RIOTU), Prince Sultan University, Riyadh 11586, Saudi Arabia; 3 Faculty of Computers and Artificial Intelligence, Benha University, Benha 13518, Egypt; 4 Department of Control and Systems Engineering, University of Technology, Baghdad 10001, Iraq; 601116@uotechnology.edu.iq

**Keywords:** mobile robot, path planning, aging-based ant colony optimization (ABACO), dynamic environment, grid-based modeling

## Abstract

Planning an optimal path for a mobile robot is a complicated problem as it allows the mobile robots to navigate autonomously by following the safest and shortest path between starting and goal points. The present work deals with the design of intelligent path planning algorithms for a mobile robot in static and dynamic environments based on swarm intelligence optimization. A modification based on the age of the ant is introduced to standard ant colony optimization, called aging-based ant colony optimization (ABACO). The ABACO was implemented in association with grid-based modeling for the static and dynamic environments to solve the path planning problem. The simulations are run in the MATLAB environment to test the validity of the proposed algorithms. Simulations showed that the proposed path planning algorithms result in superior performance by finding the shortest and the most free-collision path under various static and dynamic scenarios. Furthermore, the superiority of the proposed algorithms was proved through comparisons with other traditional path planning algorithms with different static environments.

## 1. Introduction

Robot navigation is the process of guiding a mobile robot toward the destination to perform complex tasks, such as cleaning. There are two approaches for navigation: reactive navigation and map-based navigation. In the first approach, the mobile robot has no map or any idea of where it is. The mobile robot uses random motion and acquires the information about the environment only from the contact sensor, i.e., the machine has the ability of sensing and action. On the other hand, map-based navigation is the process of creating a path for the mobile robot to move from one place to another that satisfies some criteria, such as the shortest distance and/or the lowest cost. The machine is able to sense, plan, and act, which is called path planning [1]. Several studies have been conducted to cover the problem of route planning. A grid map and improved a visible graph based on global path planning using A* algorithm was pointed out in Reference [2] and the improved A* algorithm, i.e., by considering the influence of parent node on the heuristic function of the A* algorithm, was adopted in Reference [3] for autonomous parade robot in the indoor environment. In Reference [4], the memory-efficient A* (MEA*) algorithm generated a shorter path with less time and less memory requirement when in a grid environment. A finite resistive grid was implemented in Reference [5] by converting the environment with obstacles into nodes and edges, and the optimal path was obtained by computing the least resistive path between the start and goal position. An improved version of the genetic algorithm (GA), based on special selection and the crossover function, led to a reduced computation time of GA. In addition to the shortest global path in hexagonal grid modelling, which was investigated in Reference [6], the shortest and smoothest safest path in static and dynamic environment was obtained using the Hybrid PSO-MFB algorithm and a local search, in addition to the obstacle detection and avoidance (ODA) technique, as presented in Reference [7]. Researchers in Reference [8] developed an ant colony optimization (ACO) path planner by improving the probability of selecting the optimal path to establish target attraction and proposed a wolf colony to update pheromones for an explosion proof robot (EPR). A concurrent grid-based implementation of a dynamic programming algorithm was presented in Reference [9]. In Reference [10], the flower pollination algorithm (FPA) was implemented as partially guided Q learning to solve a low convergence problem. The suggested technique implemented was a path planner for a three-wheel mobile robot. The interpolation-based path planning in a grid environment is presented in Reference [11]. Adaptive particle swarm optimization (APSO) used in Reference [12] was used to optimize the objective function of a mobile robot, which is the distance between robot to goal and obstacle. In Reference [13], the authors hybridized the artificial potential field (APF) algorithm with an enhanced genetic algorithm (EGA) to find the shortest and smoothest path for a multi-robot. An improved crossover operator based on a genetic algorithm implemented in Reference [14] was used to find the shortest and least energy of mobile robots in a static environment. The Probabilistic Roadmap (PRM) used in Reference [15] was used to construct an initial feasible short path then convert the sharp corners into a smooth corner. The fuzzy logic controller ensures the smoothest path by adjusting the heading angle. Authors in Reference [16] proposed a method to solve the path planning problem in a grid-based environment. This method included two stages: the first stage involved generation of an initial feasible path from the start point to goal point. To create this initial path, suppose the robot moves straight from its start point to its goal and turns near any obstacle it encounters in the straight line and returns to a straight line. The second stage implemented a bee colony algorithm to optimize an initial path. Additionally, a path planning-based static-grid environment using the ACO algorithm with different complexities was presented in Reference [17]. An energy-efficient routing algorithm based on information collected by a mobile agent in uneven clustering for wireless sensor networks (WSNs) is presented in Reference [18]. The PSO and GA adopt a schedule moving trajectory for the mobile sink for handling problems of hot spots in large-scale WSN, as implemented in Reference [19]. Authors in Reference [20] improve the bat algorithm in three ways by accelerating convergence processes of the bat algorithm via enhanced APF, enhancing the adaptive inertia weight and avoiding trapping in the local minimum. The shortest distance of a mobile robot in an urban area with traffic-light delay was investigated in Reference [21]. The self-organizing migration algorithm was implemented as a learning method for fuzzy cognitive map in Reference [22]. The gravitational search algorithm was adopted in a partially unknown static and dynamic environment to the final optimal path in Reference [23]. To balance between efficiency and effectiveness, the probabilistic model was used, then an estimation of the distributed algorithm and composed exhaustive search were used in Reference [24]. Hybridized Compact Form Dynamic Linearization (CFDL)-Proportional-Derivative Takagi-Sugeno Fuzzy Algorithm (PDTSFA) and Virtual Reference Feedback Tuning VRFT) proposed in Reference [25] have been used to produce a data-driven algorithm called CFDL-PDTSFA-VFET, where the parameters of CFDL-PDTSFA are optimally tuned by VFET in a model free manner.

The main objective of this work is to investigate a path planning algorithm based on aging ant colony optimization in a dynamic-grid environment as an extension of the work in [17], where the proposed methodology was implemented in a static-grid environment. 

The current paper is structured as follows. First, Section 2 introduces the problem statement and environment modelling. Section 3 presents the swarm-based optimization. The methodologies proposed for mobile robot path planning in this work are introduced in Section 4, while in Section 5, a set of simulation results are presented to demonstrate the effectiveness of the proposed methodology, as compared with other previous works. Section 6 presents the conclusion of the obtained results. 

## 2. Problem Statement and Preliminaries

Suppose the mobile robot (MR) moves from the start position (SP) to the goal position (GP) in an environment with static and dynamic obstacles to obtain certain performance criteria. The objective of a path planner is to find the optimal/near-optimal path for the mobile robot without any collision with obstacles existing in the environment.

### 2.1. Grid-Based Environment Modelling

**Figure 1:**
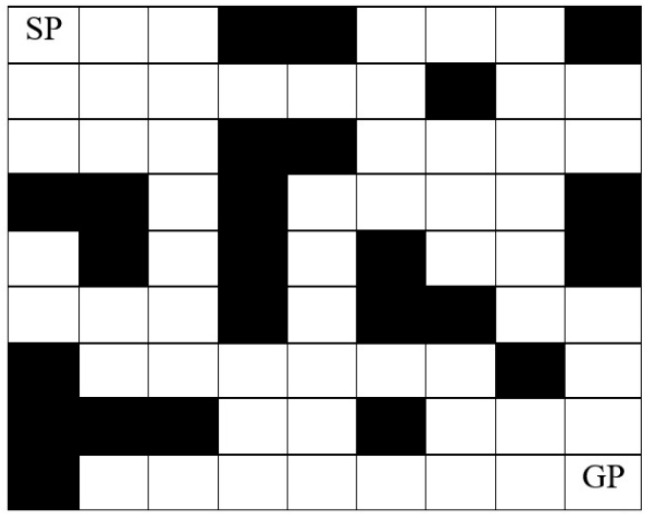
Grid-based Environment.

**Figure 2:**
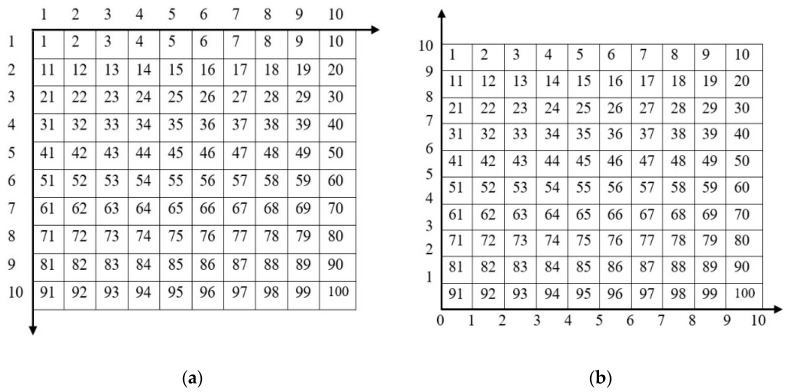
Correspondence identification types: (**a**) serial number to the grid; (**b**) serial number to (x, y) coordinates.

The first step of mobile robot path planning is to establish an environment model for the 2-Dimensional (2-D) workspace of the mobile robot. Grids are used to represent the mobile robot workspace as equal square cells. Each cell is either traversable, i.e., logic 0, or obstructed by an obstacle, i.e., logic 1, as shown in Figure 1. Each cell is identified by a unique number, called the “address”. The address of the cells is defined by two methods. The first method is the 2D grid coordinate (*r*, *c*), where the origin of the grid coordinate is the cell in the top left of the grid, with the first location having the address (1, 1). The second method is the serial number (SN) method, where the addressing of the cells begins from the left top cell and continues from left to right and top to bottom. The serial number for each cell can also be converted to its equivalent (*x*, *y*) coordinate, as shown in Figure 2. 

The mapping from the (*r*, *c*) grid to the serial number id given in Equations (1)–(3) and from the (*x*, *y*) coordinates to the serial number given in Equations (4)–(6)

(1) $$r=\lceil \frac{SN}{c_{t}}\rceil$$

(2) $$c=\{\begin{array}{cc} \left\lceil \frac{SN}{c_{t}}\right\rceil & ,R\neq 0\\ c_{t} & ,R=0 \end{array}$$

(3) $$SN=c+c_{t}\left(r-1\right)$$

(4) $$x=\{\begin{array}{cc} mod\left(\frac{SN}{x_{max}}\right) & ,\;mod\neq 0\\ x_{max}-0.5 & ,\;mod=0 \end{array}$$

(5) $$y=y_{max}+0.5-\lceil \frac{SN}{x_{max}}\rceil$$

(6) $$SN=\left(x+0.5\right)+x_{max}\left(y_{max}-\left(y+0.5\right)\right)$$

where ⌈.⌉ is the least integer function, $c_{t}$ is the number of columns of the matrix, *R* is the remainder, $x_{max}$ is the end of the abscissa, and $y_{max}$ is the end of the *y*-coordinate.

### 2.2. Performance Criteria 

**Figure 3:**
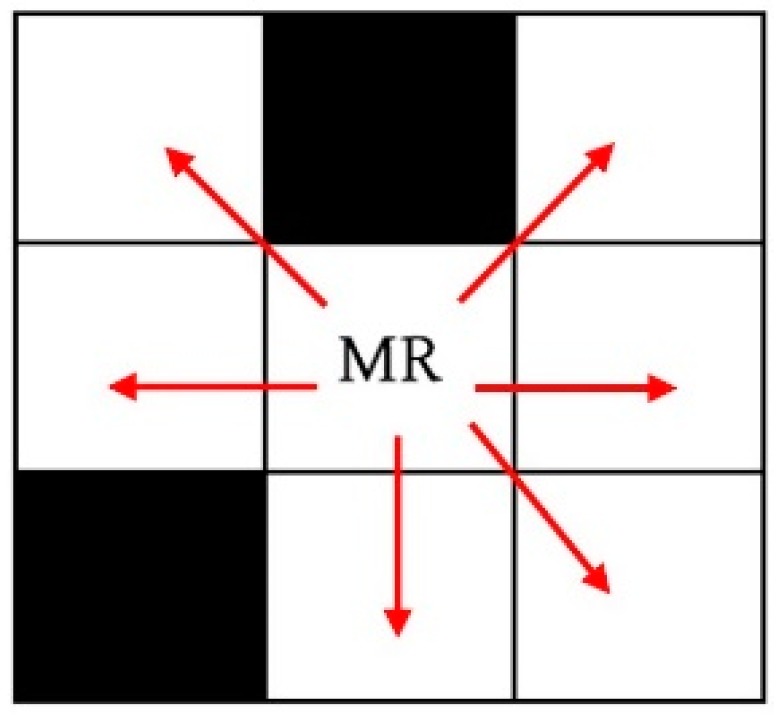
Possible path directions.

The shortest distance is the main objective for a mobile robot to move from its start position to the goal position, provided that it is a safe path, i.e., the mobile robot moves without colliding with obstacles. This criterion is given by:

(7) $$f\left(x,y\right)=\sum_{i=1}^{n-1}\sqrt{{\left(x_{i+1}-x_{i}\right)}^{2}+{\left(y_{i+1}-y_{i}\right)}^{2}}$$

(8) $$g=\frac{1}{f\left(x,y\right)+ℇ}$$

where *n* is the number of steps that the mobile robot needs to navigate toward its goal, *g* is the fitness of the solution, and ℇ is a small number, e.g., ℇ=0.001, used to prevent a division by 0. In every step, the mobile robot can move from its current location to another one of its surrounding free cells, as shown in Figure 3. 

The optimization problem can be defined as:

*“Find the shortest distance between the start point (SP) and the goal point (GP), such that the above criteria*g*(Equation (8)) is maximized”*.

### 2.3. Obstacles Movement

In this case, the obstacles change their location continuously at each time step, and, in dynamic environments, the position of the obstacle ($x_{obs}$, $y_{obs}$) is updated according to,

(9) $$x_{obs}=x_{obs}+v_{obs}\times \cos\theta_{obs}$$

(10) $$y_{obs}=y_{obs}+v_{obs}\times \sin\theta_{obs}$$

where $v_{obs}$ is the velocity of obstacles and $\theta_{obs}$ is the slope of the linear motion.

## 3. Swarm-Based Optimization Algorithms 

### 3.1. Ant Colony Optimization 

Swarm intelligence is based on nature-inspired behavior and is successfully applied to optimize problems in a variety of applications. The ant colony optimization (ACO) algorithm is a stochastic-based optimization technique that replicates the behavior of real ants when searching for food, invented by Dorigo [26]. It discovers the shortest route from an ant nest to food places through the interchange of information collaboration [27]. The ants move along the same path by following one another. This is because every ant leaves a chemical substance called pheromone while moving on the path. The other ants sense the intensity of the pheromone and follow the path with a higher concentration of pheromone. This is their tactic to find an optimized path. Initially, the ants wander randomly to find their way to the destination. On their back tour, the ants sense the pheromone intensity and choose the path with a higher concentration of pheromone. The pheromone evaporates with time and hence the concentration of pheromone would be higher along the shortest path as the time taken to cover the shortest path would be minimal as compared to other paths. Hence, almost every ant would be attracted by the higher intensity of the pheromone along the shortest path and selects the optimized path. The philosophy of ant behavior can be summarized as follows.

#### 3.1.1. Ant Searching Behavior

For the *k*th ant at position *i* to move to the next node *j*, the following probability formula is used [28,29]:

(11) $$P_{ij}\left(k\right)=\frac{\tau_{ij}^{\alpha }\left(k\right)*\eta_{ij}^{\beta }\left(k\right)}{\sum_{k=1}^{S}\tau_{ij}^{\alpha }*\eta_{ij}^{\beta }}$$

where *α* and *β* are the degrees of importance of pheromones and heuristic function, respectively, $\tau_{ij}$ is the pheromone concentration on the path between *i* and *j*, and $\eta_{ij}$ is the heuristic function, i.e., which is equivalent to the reciprocal of the distance between the *i* and *j* positions.

#### 3.1.2. Path Retracing and Pheromone Updating

After ants complete their tour, the pheromone trial values are updated according to [30]:

(12) $$\tau_{ij}\left(t+1\right)=\left(1-\rho \right)\tau_{ij}\left(t\right)+\Delta \tau_{ij}$$

where ρ is the pheromone decay parameter range ∈ (0, 1) to mimic the evaporation of the pheromone and $\Delta \tau_{ij}$ is the amount of pheromones added by all the ants.

(13) $$\Delta \tau_{ij}=\sum_{k=1}^{N}\Delta \tau_{ij}^{k}$$

(14) $$\Delta \tau_{ij}^{k}=\frac{Q}{L_{k}}$$

where $\Delta \tau_{ij}^{k}$ is the amount of pheromones added by the *k*th ant, Q is the pheromone update constant, N is the total number of ants in the nest, and $L_{k}$ is the length of the path traveled by the *k*th ant.

### 3.2. Aging-Based Ant Colony Optimization (ABACO)

**Figure 4:**
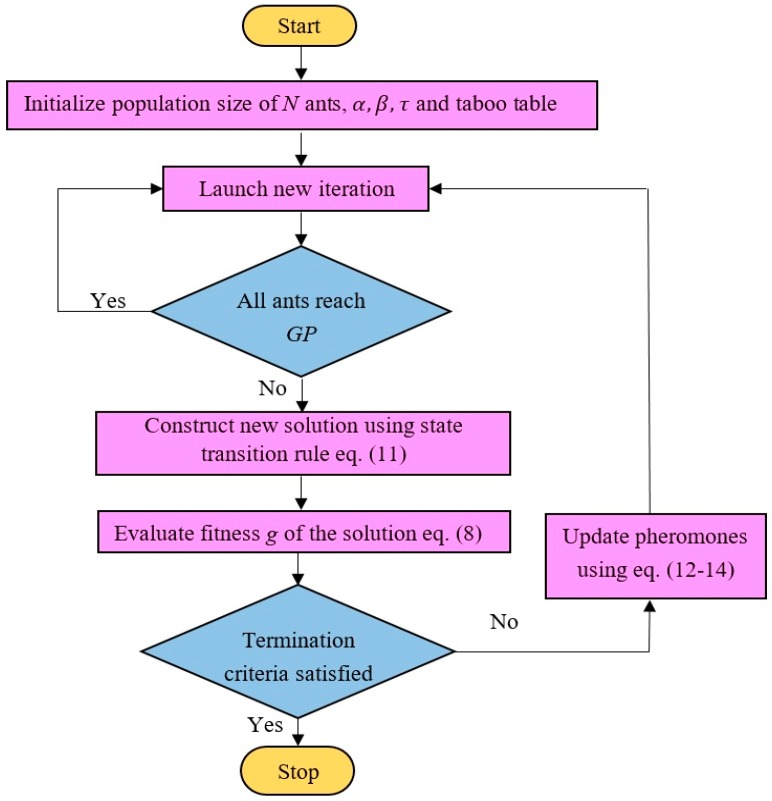
Flow chart of an aging-based ant colony optimization (ABACO) algorithm.

In standard ACO, the amount of pheromone deposits by the ants is assumed to be constant. Pheromone control can be used to reduce the influence of non-optimal solutions and encourages the exploration of new paths that are near-optimal or optimal. Bad experiments can also be reduced by controlling the amount of pheromones for each ant according to its age. Ant aging is based on the rationale that old ants are less successful in locating the optimal paths, since they take a longer time to reach their destination. Both aging and evaporation encourage the discovery of new paths that are previously non-optimal. The amount of pheromone released by each ant is given by Equation (13), and Equation (14) indicates that $\Delta \tau_{ij}\left(k\right)$ varies according to the age of the ant. The inclusion of the age of ants is suggested by allocating random values of *Q* to different ants. This is reflected in the updated pheromones in Equation (14), being different for each ant. Hopefully, the assignment process for the random values of *Q* is done in such a way that the ant with the shortest path, i.e., the younger ant, gets a higher value of *Q*, which in turn has a higher value of pheromone. In contrast, the ant with the longest path, i.e., the old ant, gets a lower pheromone update, which means it is assigned a lower value of *Q*. Figure 4 shows the flow chart of the aging-based ant colony optimization (ABACO) algorithm.

The optimization problem of finding the shortest distance will be solved by ABACO, which will be explained next in this paper.

### 3.3. Algorithmic Iteration Concepts and Feasible Solutions 

**Figure 5:**
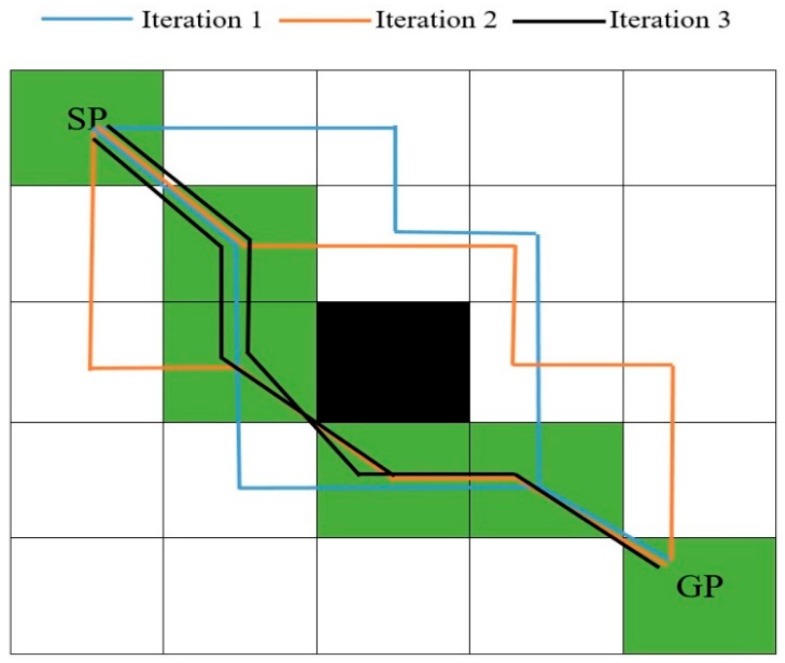
Solution construction in an ant colony optimization (ACO)-based path planning algorithm.

In the ABACO algorithm, the solution from the start point to the goal point through various layers in between represents one complete iteration. Each ant can select one cell in each layer in accordance with the state transition rule given by Equation (11). Figure 5 depicts three iterations with two ants; each ant constructs its solution, i.e., a series of nodes selected by each ant from the start point (SP) to the goal point (GP). The best path is obtained after a certain number of iterations with a higher amount of pheromone, i.e., the shaded cells, i.e., the green cells, represent the best solution.

## 4. Proposed Path Planning Algorithm

**Figure 6:**
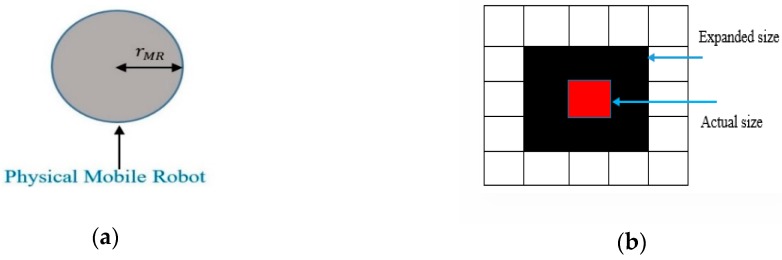
Expanding obstacles size corresponding to the mobile robot size: (**a**) physical robot; (**b**) grid shaped obstacle.

**Figure 7:**
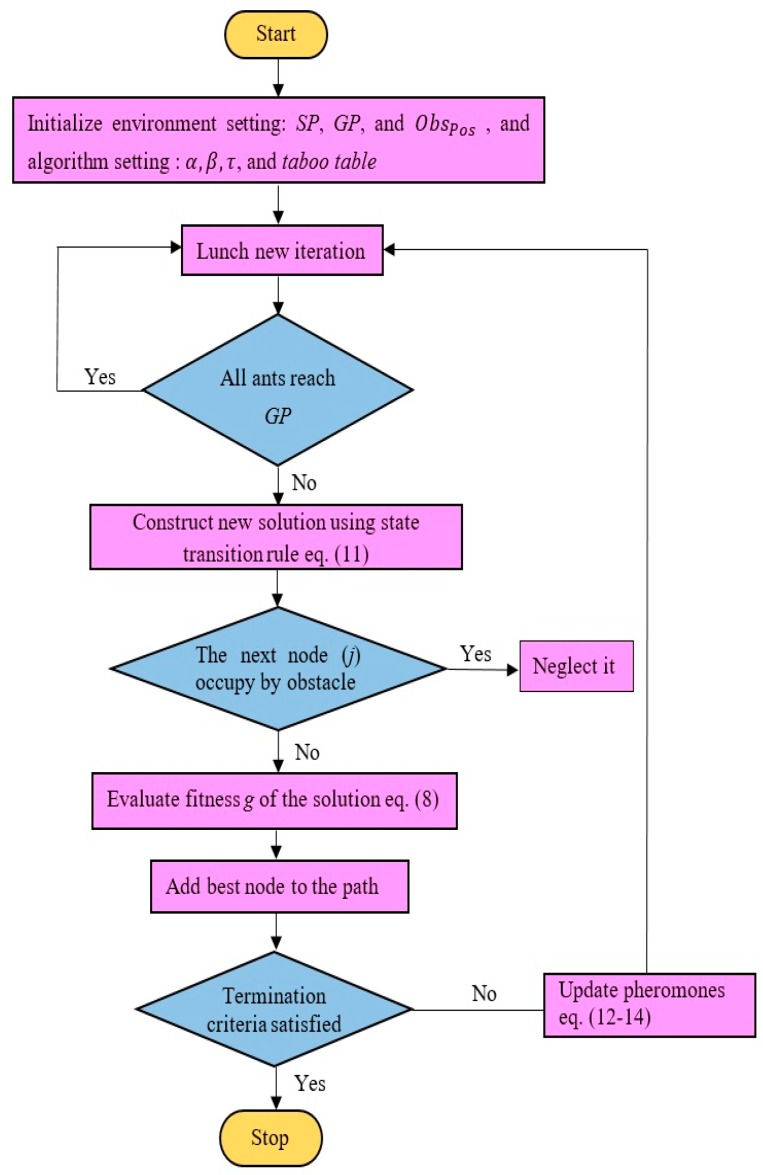
Flowchart of mobile robot path planning in a static environment using ABACO algorithm.

**Figure 8:**
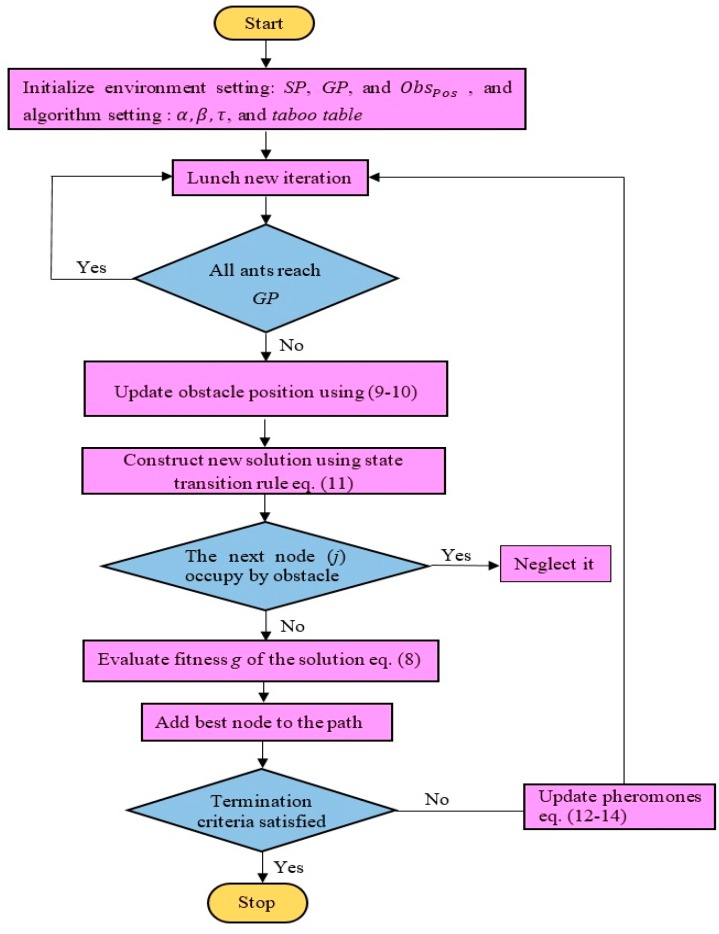
Flowchart of mobile robot path planning in a dynamic environment using ABACO algorithm.

Since the mobile robot is a physical body, the obstacles are expanded by the radius of the mobile robot $r_{MR}$, i.e., to take into account the actual size of the mobile robot, and then the mobile robot is considered as a point. Figure 6 illustrates the expansion of the obstacle size. The flowchart for a mobile robot path planning in grid static and dynamic environments using ABACO is shown in Figure 7 and Figure 8, respectively.

## 5. Simulation Results

### 5.1. Effect of Design Parameters on ABACO

This section presents the influence of design parameters: number of iteration, ants, and evaporation factor (ρ) on a global search. The results were applied to 18 × 18 (m^2^) and 28 × 28 (m^2^), as given below:

#### 5.1.1. Number of Iterations 

**Figure 9:**
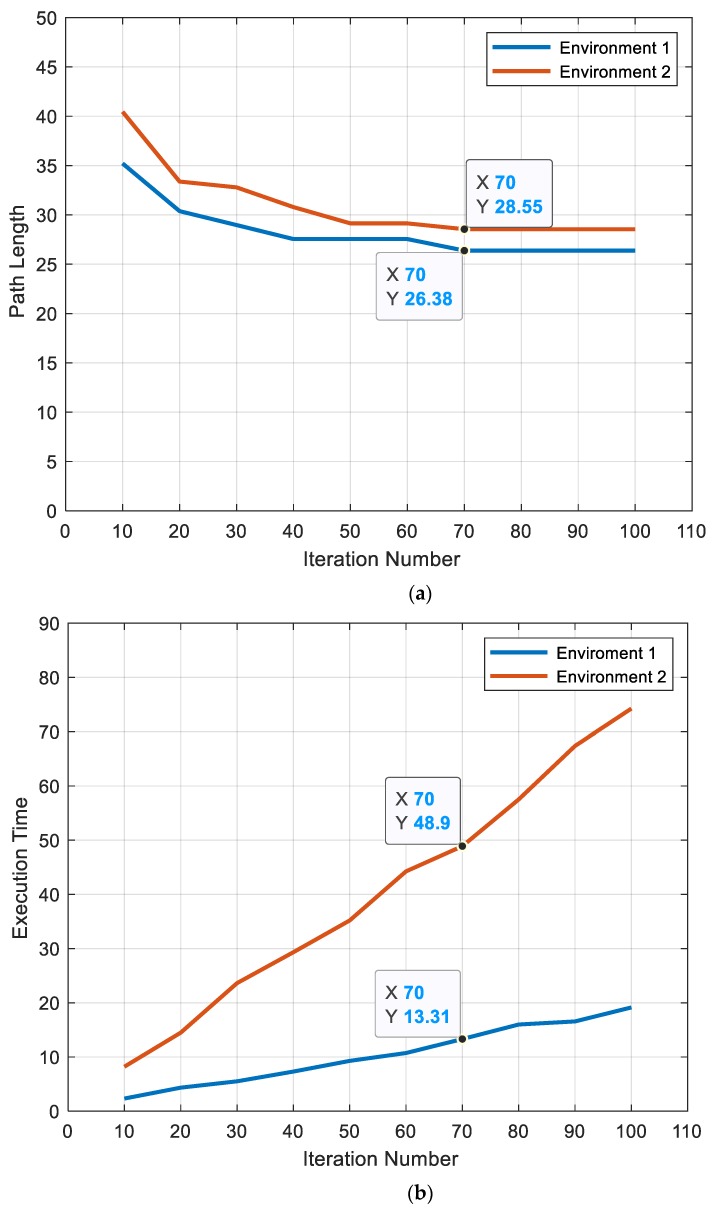
Effect of the number of iterations on (**a**) path length and (**b**) execution time for environment 1 and environment 2.

From Figure 9a, it is evident that the algorithm found an optimal path with an increase in the total number of iterations. As shown in Figure 9a, at iteration (70), the path lengths were (26.3848 m) and (28.5563 m) for 18 × 18 (m^2^) and 28 × 28 (m^2^) map sizes, respectively. Additionally, the whole time required is increased as shown in Figure 9b.

#### 5.1.2. Number of Ants

**Figure 10:**
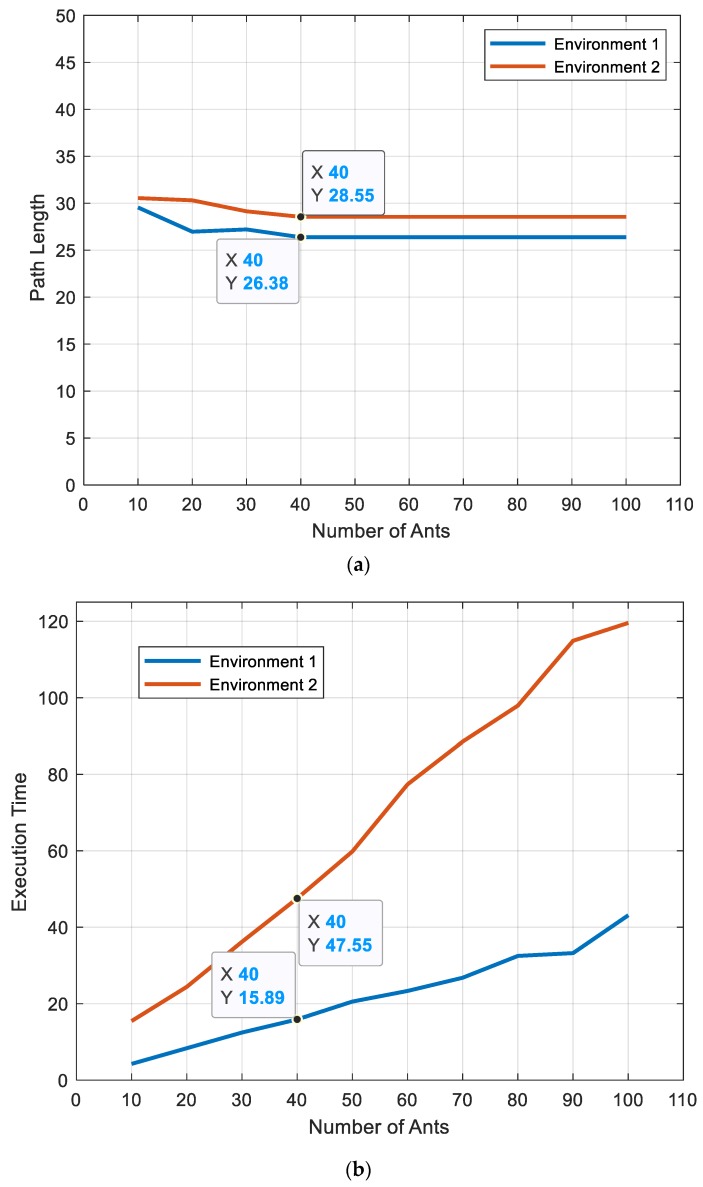
Effect of the number of ants on (**a**) path length and (**b**) execution time for environment 1 and environment 2.

By changing the total number of ants in the colony from (10) to (100), as shown in Figure 10a, the best path lengths obtained for 18 × 18 (m^2^) map and 28 × 28 (m^2^) were (26.3848 m) and (28.5563 m), respectively, with 40 ants in the colony, as shown in Figure 10a,b. In addition, the total time required increased, as shown in Figure 10b.

#### 5.1.3. Evaporation Factor (ρ)

**Figure 11:**
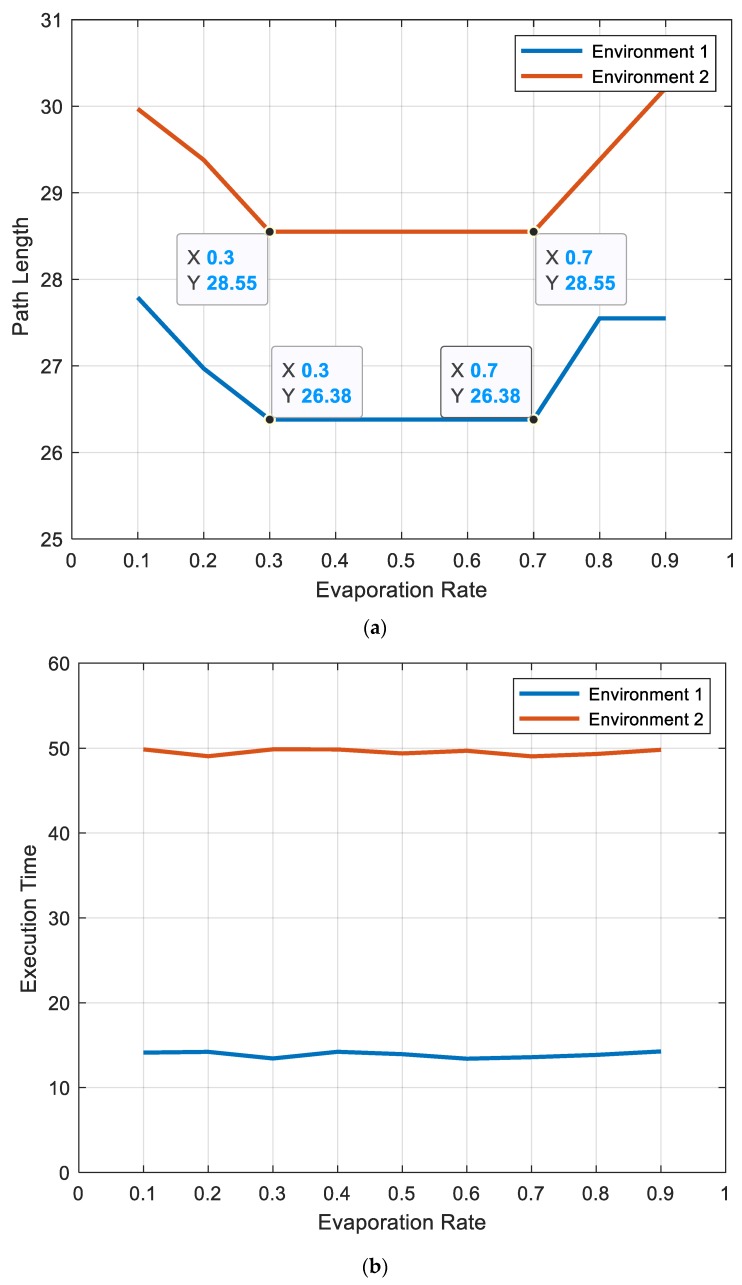
Effect of the evaporation factor (*ρ*) on (**a**) path length and (**b**) execution time for environment 1 and environment 2.

The best value of evaporation factor (ρ) was between (0.3) and (0.7) for all dimensions of the searching space, as shown in Figure 11a, and had no effect on the computation time, as depicted in Figure 11b.

### 5.2. Static Environment with Grid-Based Modeling

The ABACO algorithm applied for path planning plans the entire path from the start point up to the endpoint in one complete iteration. 

#### 5.2.1. Grid-Based Static Environment 1

**Figure 12:**
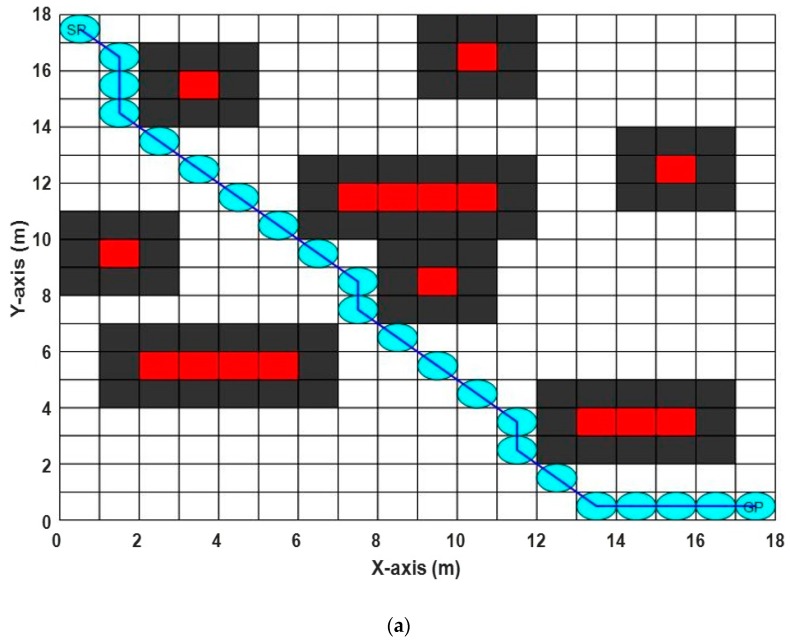

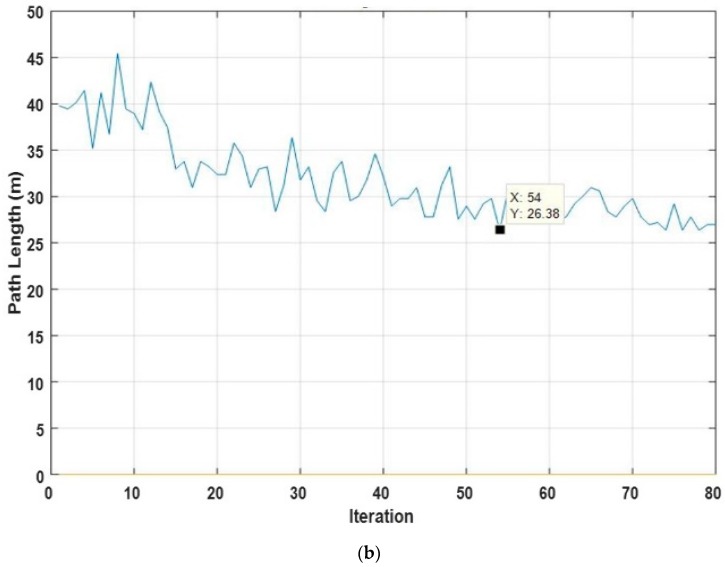
The results of the proposed ABACO-based path planning algorithm in grid-based static environment 1, (**a**) the best path achieved, (**b**) the convergence curve of the proposed ABACO based path planning algorithm.

In this experiment, the size of the search space was (18 × 18 m^2^) per grid cell, and there were eight obstacles with different sizes and locations, as shown in Table 1. After executing the ABACO algorithm 10 times, the shortest path length was (26.3848 m) at iteration (54) with different execution times, ranging from (0.2255 min) to (0.3057 min). The best path obtained (see Figure 12a) and the convergence curve for this environment (See Figure 12b) are shown in Figure 12a,b respectively.

#### 5.2.2. Grid-Based Static Environment 2

In this experiment, the size of the search space was (28 × 28 m^2^) per grid cell, and there were 14 obstacles with different sizes and locations, as shown in Table 2.

**Figure 13:**
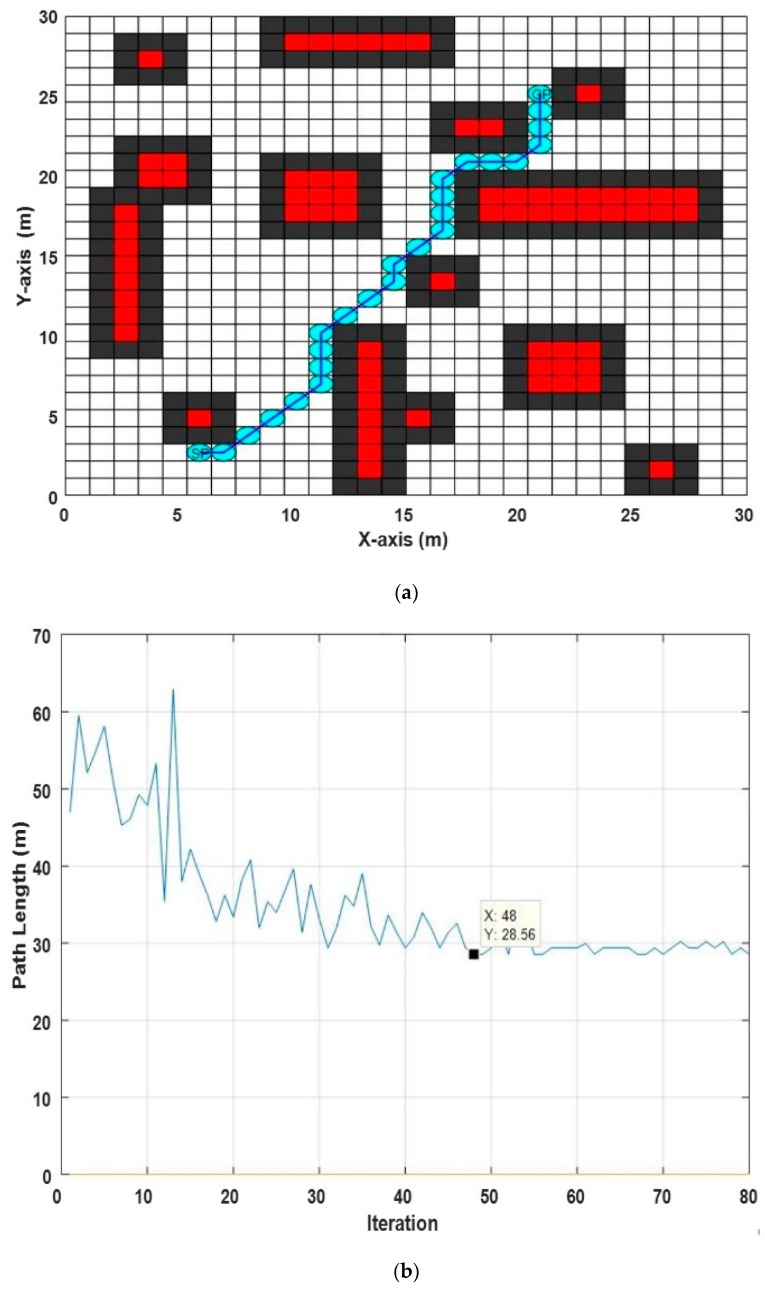
The results of the proposed ABACO-based path planning algorithm in grid static environment 2, (**a**) the best path obtained, (**b**) the convergence curve of the proposed ABACO based path planning algorithm.

After running ABACO 10 times, the shortest path length was found to be (28.5563 m) at iteration (48), with different execution times ranging from (0.8231 min) to (0.875 min). The best path obtained (see Figure 13a) and the convergence curve for this environment (see Figure 13b) are shown in Figure 13a,b, respectively.

### 5.3. Dynamic Environment with Grid-Based Modeling

#### 5.3.1. Grid-Based Dynamic Environment 1

This environment consisted of three moving obstacles, defined in Table 3: The starting point (SP) at the location (5, 3) grid coordinate, the goal point (GP) at the (15, 15) grid coordinate, and the $r_{MR}$ at (0.5).

**Figure 14:**
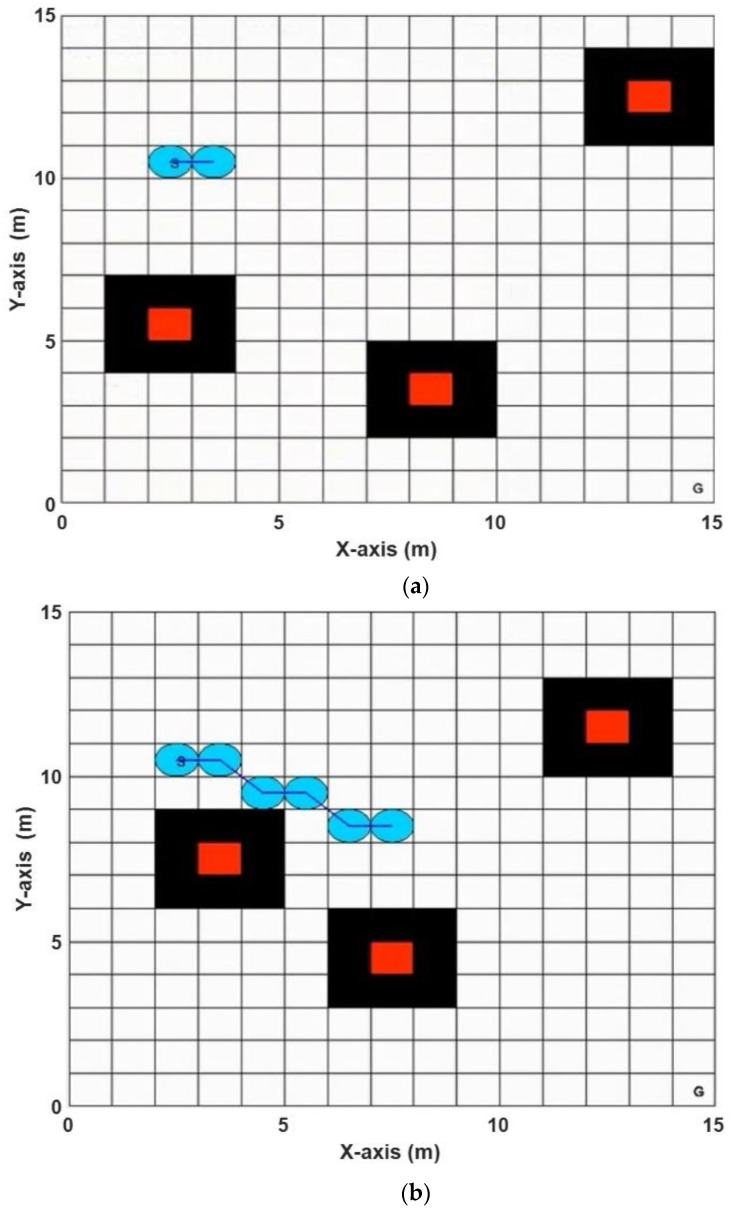

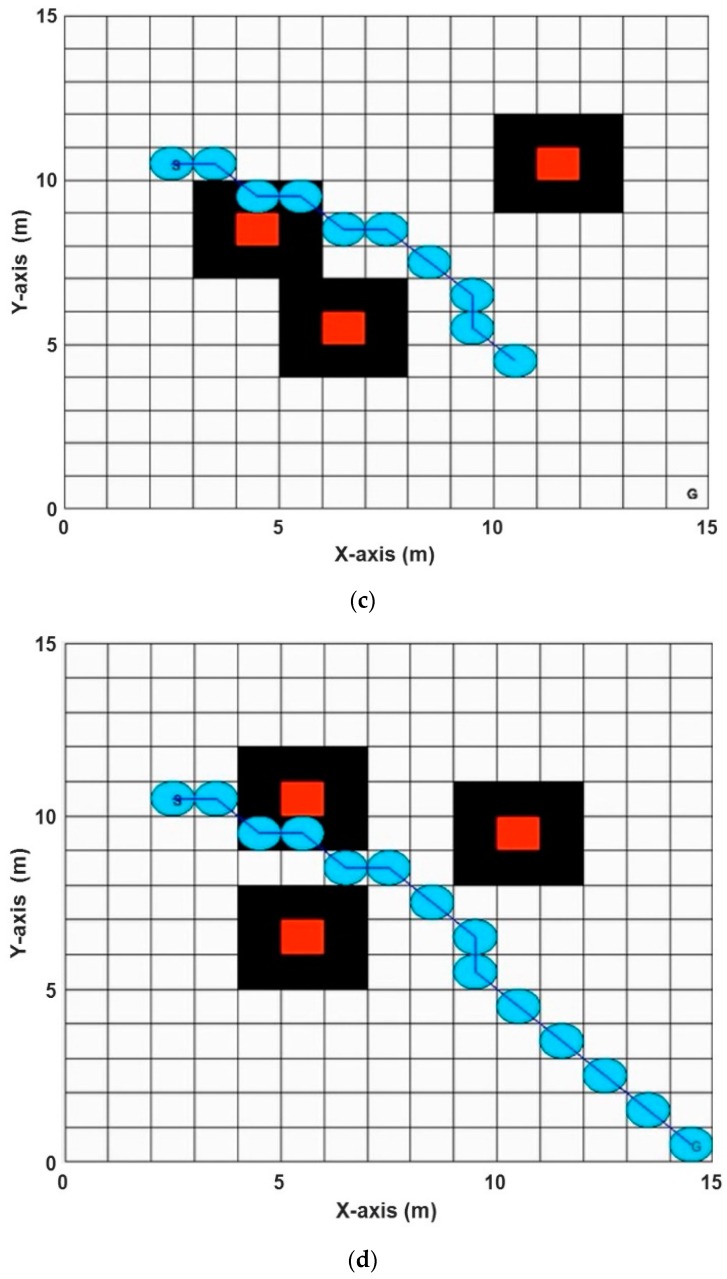
The best path achieved within the ABACO grid-based dynamic environment 1, (**a**) the mobile robot starts from its starting position, (**b**) the mobile robot is moving toward its goal position, (**c**) the mobile robot is at its midway and is avoiding the obstacles, (**d**) the mobile robot is approaching its goal position.

Table 4 shows the obtained solutions and their corresponding computation times using the ABACO algorithm, where the optimize fitness function is defined as in Equation (8). From Table 4, it can be concluded that run number (1) gave the best results with the shortest path length of (19.7279 m) and computation time (1.3876 min) (see bold values). Figure 14 shows the planned path of the mobile robot while avoiding three dynamic obstacles with different velocity, and each obstacle moved according to Equations (9) and (10), then the mapping in the new position from (*x*, *y*) coordinated to the corresponding (*r*, *c*) grid coordinate.

#### 5.3.2. Grid-Based Dynamic Environment 2

This environment included five moving obstacles with initial locations and orientation, as defined in Table 5: The start point (SP) at the location (4, 15) grid coordinate, the goal point (GP) at the (17, 5) grid coordinate, and the $r_{MR}$ is (0.5).

**Figure 15:**
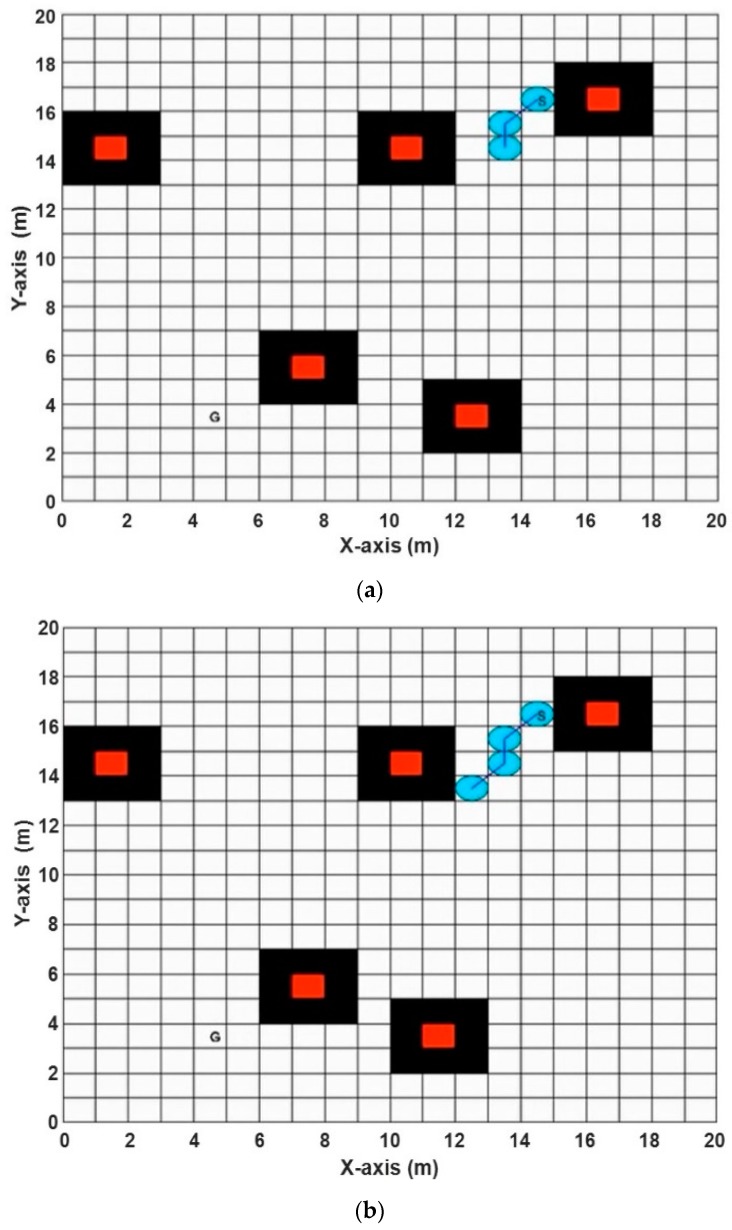

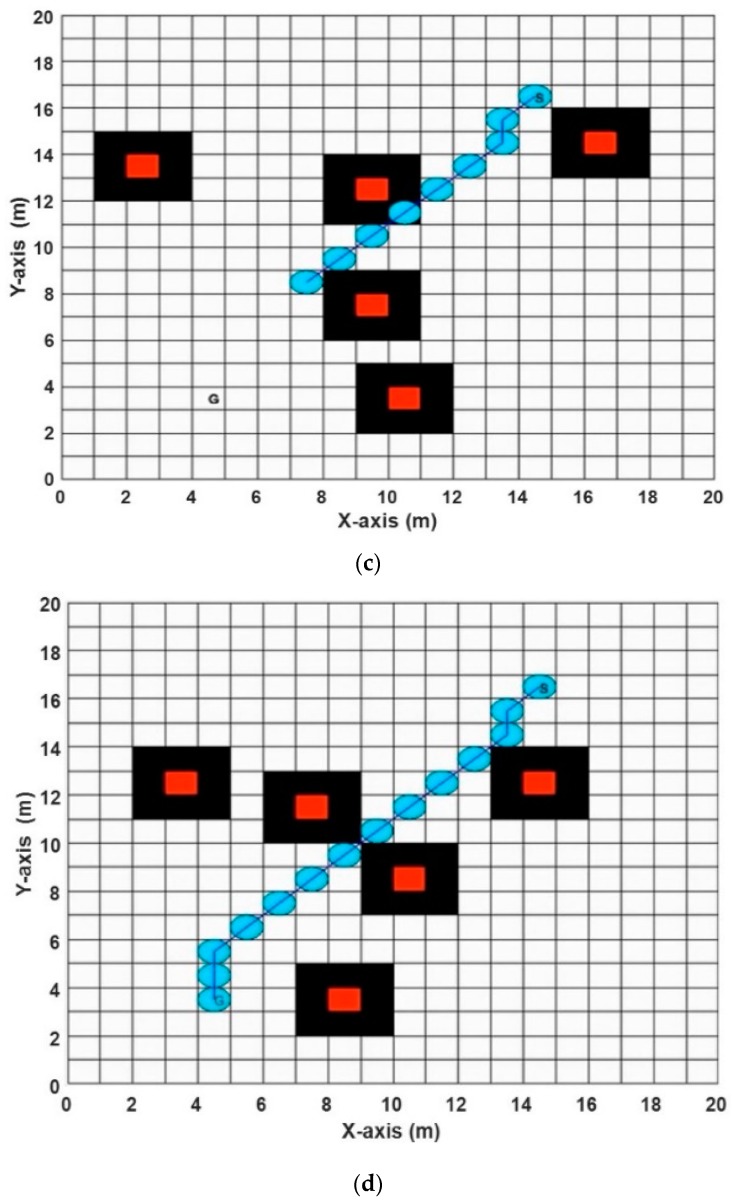
The best path achieved using the ABACO –based path planning algorithm in grid-based dynamic environment 2, (**a**) the mobile robot starts from its starting position, (**b**) the mobile robot is moving toward its goal position, (**c**) the mobile robot is at its midway and is avoiding the obstacles, (**d**) the mobile robot is approaching its goal position.

Table 6 shows the achieved solutions and computation times for 10 executions using the ABACO algorithm. The best results are found at run number (3) with the shortest path length of (23.7279 m) and computation time (7.4359 min) (see bold values). Figure 15 shows the planned path of the mobile robot while avoiding five dynamic obstacles with different velocity and direction. Each obstacle moved according to Equations (9) and (10), then the mapping of the new position from (*x*, *y*) coordinated to the corresponding (*r*, *c*) grid coordinate.

### 5.4. Comparison with Other Previous Works

In order to show the efficiency of the proposed algorithms, these algorithms were compared with other algorithms in different researches. For a fair comparison, the same assumptions, including the map dimension, number of obstacles, and consideration of the mobile robot as a single point, were used. 

**Figure 16:**
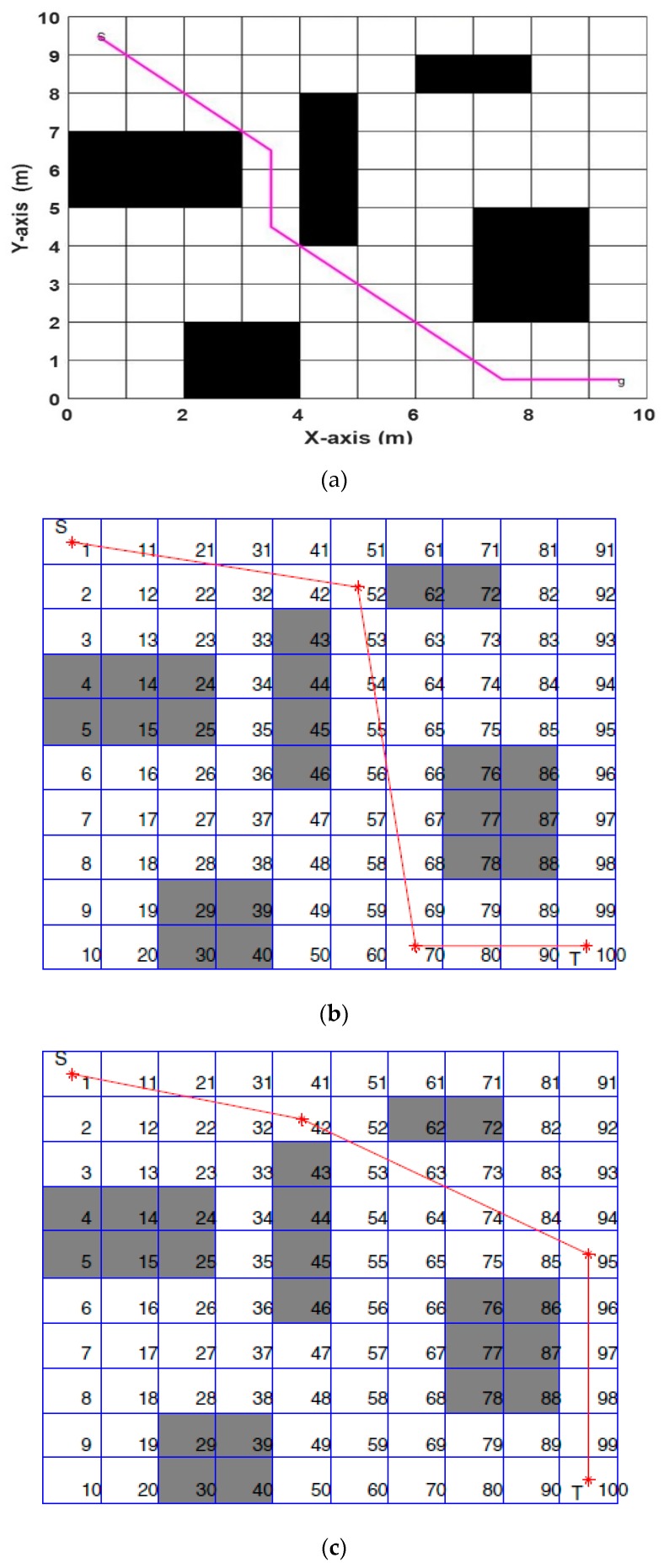

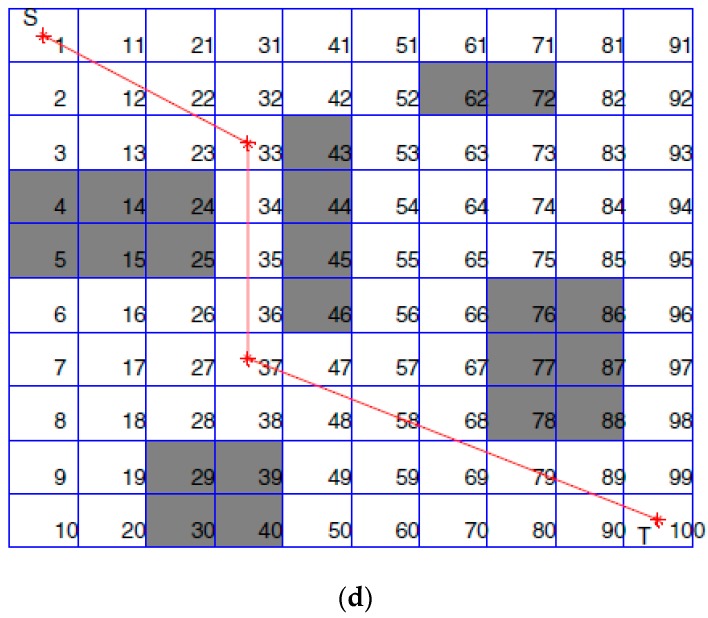
The best path achieved by (**a**) aging-based ant colony optimization (ABACO), (**b**) the genetic algorithm (GA) [31], (**c**) pattern search (PS) [31], and (**d**) particle swarm optimization (PSO) [31] (Map 1).

**Figure 17:**
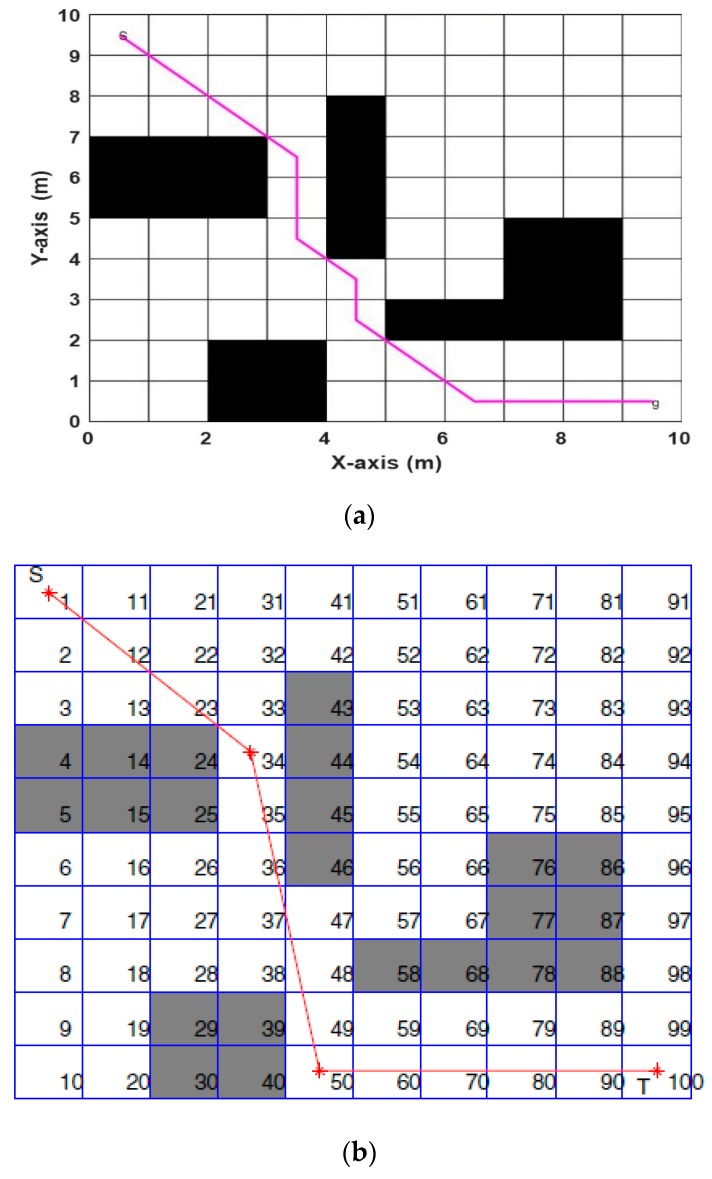

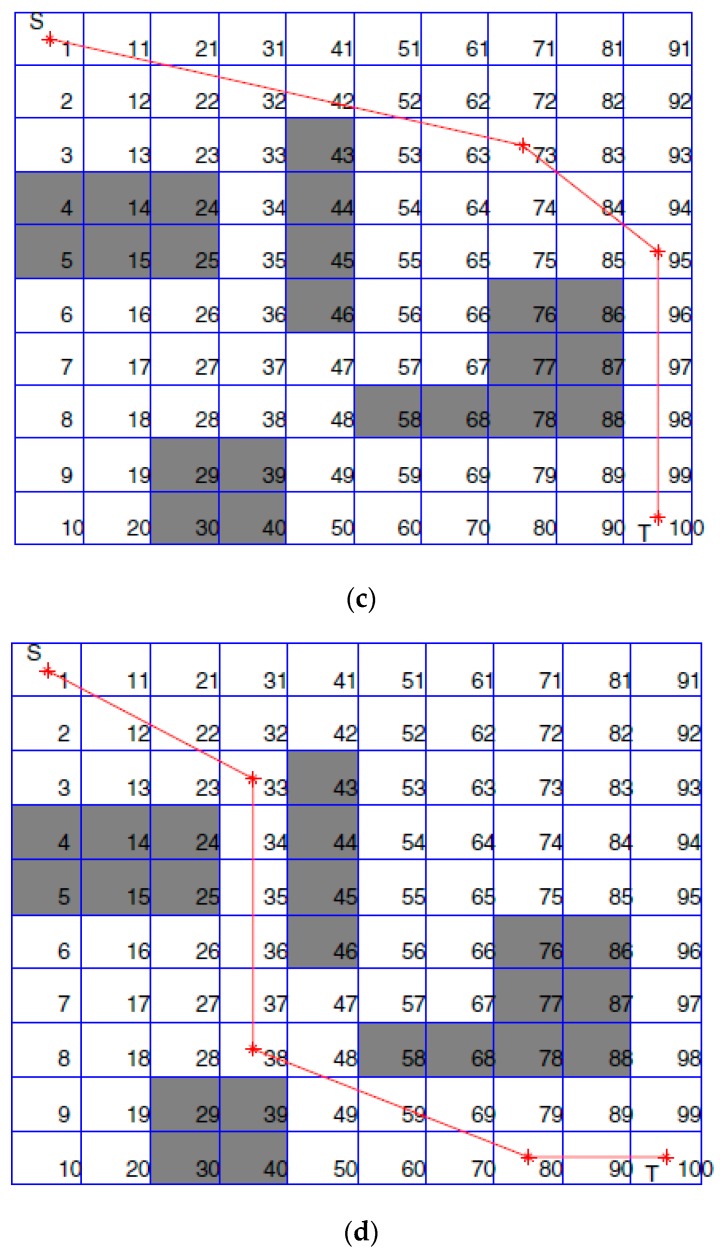
The best path achieved by (**a**) ABACO, (**b**) GA [31], (**c**) PS [31], and (**d**) PSO [31] (Map 2).

Now, we discuss the Performance Evaluation of the ABACO based path planning algorithm in a Static Grid environment. In [31], three algorithms were implemented to solve path planning in a static grid for two environments. These algorithms were the pattern search (PS) algorithm, the genetic algorithm (GA), and particle swarm optimization (PSO). Figure 16 represents the first environment with five static obstacles, while the second environment consists of four static obstacles, as shown in Figure 17. The proposed ABACO algorithm was applied to both environments and the comparison results between all these algorithms are listed in Table 7.

**Figure 18:**
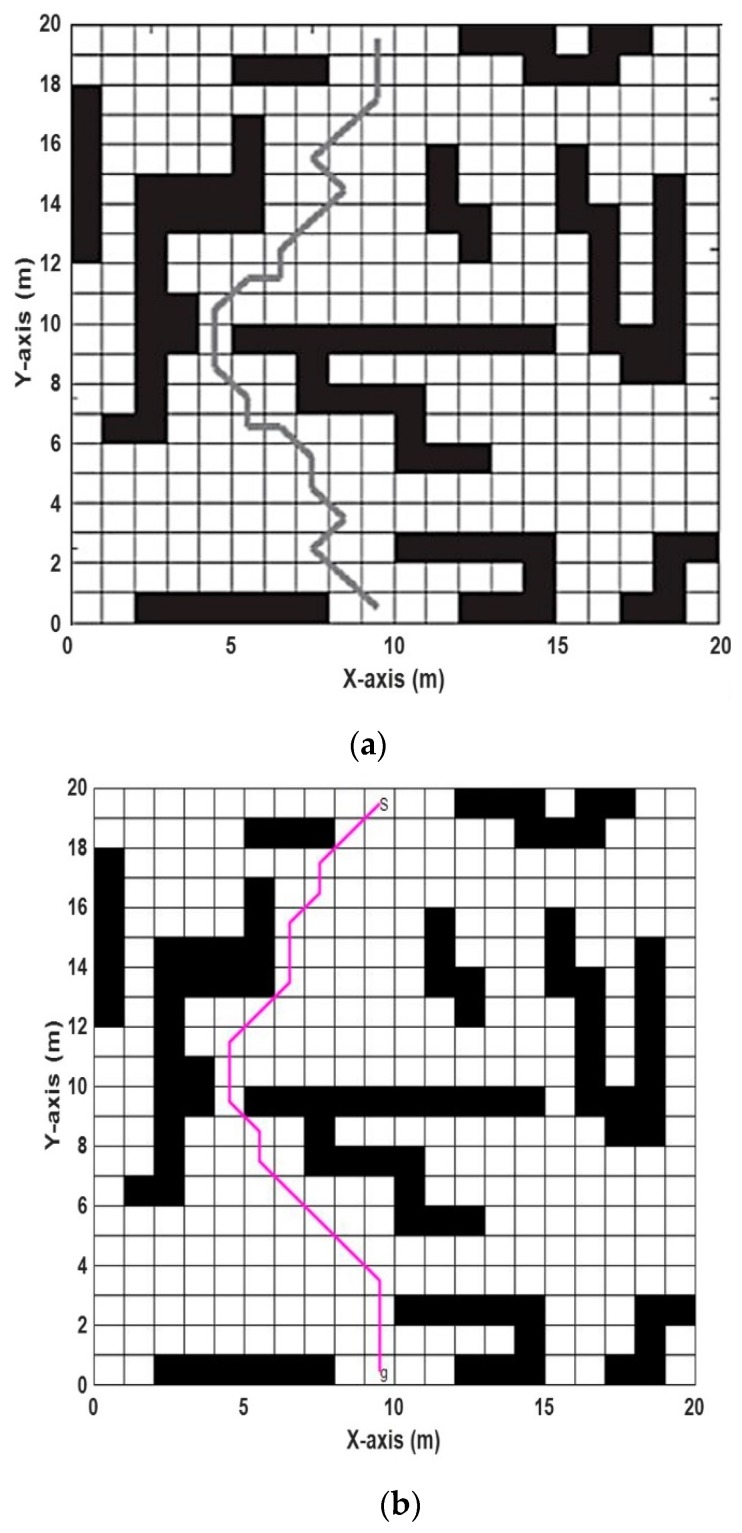
(20 × 20) grid using (**a**) the ABC algorithm and (**b**) the ABACO algorithm.

Table 7 shows that the shortest path for the first and second environments was obtained using the ABACO algorithm, which were (13.8995) and (14.4853), respectively (see bold values). The improvement ratio (IR) of the proposed algorithm compared with other algorithms is calculated by the following relation:

(15) $$IR=\left|\frac{PA-OA}{OA}\right|\times 100\%$$

where PA is the proposed algorithm and OA is the other algorithms. The IR for the ABACO, compared with algorithms listed in Table 7, is presented in Table 8, in terms of the shortest distance. In [16], the case study was performed using artificial bee colony (ABC) optimization algorithms. The environment consisted of a 20 × 20 m^2^ grid with different obstacles. The start position was from the middle-top of the grid to the middle-bottom of the grid as a goal point. The ABC algorithm was applied to obtain the shortest distance. The ABACO was applied to the same environment and obtained the results, as shown in Figure 18. The best path obtained using ABC, as shown in Figure 18a, was (25.9706), while the path obtained using ABACO algorithm, as shown in Figure 18b, was 23.1421 m for the same environment. The improvement ratio (IR) for the ABACO over the ABC was (10.89%). 

## 6. Conclusions

The path planner based on ABACO and grid-based modelling is proposed in this paper to find the optimal/near-optimal path of the mobile robot in static and dynamic environments. The age of the ant was taken into consideration to produce a new kind of ACO optimization. which is exploited in the design of mobile robot path planning. Grid-based modelling was found to be less flexible (hard of implementation) in dynamic environments as compared to free-space-based modelling. However, it easily modelled the mobile robot and the obstacles in the 2D-environment. When the proposed ABACO algorithm was applied to mobile robot path planning, it was found that the proposed ABACO algorithm outperformed the standard ACO algorithm in terms of the shortest path. Based on the comparison results, the ABACO algorithm is superior to GA, PSO, and ABC. Finally, to overcome the unused space inside the cell using grid-based modelling, a future work will be conducted by increasing the resolution of the grid, which will supposedly help in reducing the waste of space of the individual cells and result in a smoother path from the start to end points.

## Figures and Tables

**Table 1:** Grid-based static environment 1 setting.

Point Type	Position (row, col)
SP	(1, 1)
GP	(18, 18)
$r_{MR}$	0.5
${\mathrm{Obs}}_{1}$	(3, 4)
${\mathrm{Obs}}_{2}$	(2, 11)
${\mathrm{Obs}}_{3}$	(7, 8:11)
${\mathrm{Obs}}_{4}$	(9, 2)
${\mathrm{Obs}}_{5}$	(10, 10)
${\mathrm{Obs}}_{6}$	(6, 16)
${\mathrm{Obs}}_{7}$	(13, 3:6)
${\mathrm{Obs}}_{8}$	(15, 14:16)

**Table 2:** Grid-based static environment 2 setting.

Point Type	Position (row, col)
SP	(26, 6)
GP	(5, 20)
$r_{MR}$	0.5
${\mathrm{Obs}}_{1}$	(3, 4)
${\mathrm{Obs}}_{2}$	(2, 10:15)
${\mathrm{Obs}}_{3}$	(5, 22)
${\mathrm{Obs}}_{4}$	(7, 17:18)
${\mathrm{Obs}}_{5}$	(9:10, 4:5)
${\mathrm{Obs}}_{6}$	(10:12, 10:12)
${\mathrm{Obs}}_{7}$	(12:19, 3)
${\mathrm{Obs}}_{8}$	(16, 16)
${\mathrm{Obs}}_{9}$	(20:27, 13)
${\mathrm{Obs}}_{10}$	(24, 15)
${\mathrm{Obs}}_{11}$	(20:22, 20:22)
${\mathrm{Obs}}_{12}$	(24, 6)
${\mathrm{Obs}}_{13}$	(27, 25)
${\mathrm{Obs}}_{14}$	(11:12, 18:26)

**Table 3:** Grid-based dynamic environment 1 setting.

${\mathrm{Obs}}_{\mathrm{no}.}$	${\mathrm{Obs}}_{\mathrm{Pos}}\left(row,\;col\right)$	${\mathrm{Obs}}_{\mathrm{Pos}}\left(x,\;y\right)$	$V_{Obs}$ (m/s)	$\theta_{\mathrm{Obs}}$
${\mathrm{Obs}}_{1}$	(10, 3)	(2.5, 5)	0.5	60°
${\mathrm{Obs}}_{2}$	(5, 15)	(14.5, 13.1)	0.39	230°
${\mathrm{Obs}}_{3}$	(12, 10)	(9.1, 3.5)	0.36	0°

**Table 4:** Simulation results for the grid-based dynamic environment 1 using the ABACO algorithm.

Run	Path Length (m)	Fitness	Computation Time (min)
**1**	**19.7279**	**0.050689632**	**1.3876**
2	20.5563	0.048646886	3.0373
3	20.3137	0.0492278610	1.5335
4	19.7279	0.0506896324	1.3946
5	21.7279	0.0460237758	1.4395
6	20.5563	0.0486468868	1.6428
7	19.7279	0.0506896324	1.4544
8	19.7279	0.0506896324	1.3837
9	20.5563	0.0486468868	1.8156
10	19.7279	0.0506896324	1.4833

**Table 5:** Grid-based dynamic environment 2 setting.

${\mathrm{Obs}}_{\mathrm{no}.}$	${\mathrm{Obs}}_{\mathrm{Pos}}\left(row,\;col\right)$	${\mathrm{Obs}}_{\mathrm{Pos}}\left(x,\;y\right)$	$V_{Obs}$ (m/s)	$\theta_{\mathrm{Obs}}$
${\mathrm{Obs}}_{1}$	(5, 2)	(1.5, 15.1)	0.23	−45°
${\mathrm{Obs}}_{2}$	(5, 12)	(11.3, 15.6)	0.43	230°
${\mathrm{Obs}}_{3}$	(16, 7)	(6.5, 4.5)	0.47	45°
${\mathrm{Obs}}_{4}$	(17, 14)	(13, 3)	0.34	180°
${\mathrm{Obs}}_{5}$	(3, 18)	(17.1, 17.1)	0.36	250°

**Table 6:** Simulation results for the grid-based dynamic environment 2 using the ABACO algorithm.

Run	Path Length (m)	Fitness	Computation Time (min)
1	23.9706	0.041717770	8.1258
2	23.9706	0.041717770	8.2659
**3**	**23.7279**	**0.042144479**	**7.4359**
4	23.7279	0.042144479	8.7085
5	25.9706	0.038505078	8.7177
6	23.7279	0.042144479	9.9965
7	25.9706	0.038505078	8.8647
8	23.9706	0.041717770	8.2728
9	25.3848	0.039393652	8.5407
10	23.7279	0.042144479	9.3426

**Table 7:** Performance comparison of the proposed ABACO and the algorithms of [31].

	Algorithm	GA	PS	PSO	ABACO
Environment 1	Path Length	17.8051	17.3103	16.9362	13.8995
Environment 2	Path Length	18.4410	17.7430	17.7001	14.4853

**Table 8:** Improvement ratio (IR) of the ABACO and algorithms in [31].

IR for ABACO with:	GA	PS	PSO
Environment 1	21.9%	19.7%	17.9%
Environment 2	21.45%	18.3605%	18.1626%
